# Validity of the Catapult ClearSky T6 Local Positioning System for Team Sports Specific Drills, in Indoor Conditions

**DOI:** 10.3389/fphys.2018.00115

**Published:** 2018-04-04

**Authors:** Live S. Luteberget, Matt Spencer, Matthias Gilgien

**Affiliations:** Department of Physical Performance, Norwegian School of Sport Sciences, Oslo, Norway

**Keywords:** kinematics, position, instantaneous speed, accuracy, performance analyses, physical demands

## Abstract

**Aim:** The aim of the present study was to determine the validity of position, distance traveled and instantaneous speed of team sport players as measured by a commercially available local positioning system (LPS) during indoor use. In addition, the study investigated how the placement of the field of play relative to the anchor nodes and walls of the building affected the validity of the system.

**Method:** The LPS (Catapult ClearSky T6, Catapult Sports, Australia) and the reference system [Qualisys Oqus, Qualisys AB, Sweden, (infra-red camera system)] were installed around the field of play to capture the athletes' motion. Athletes completed five tasks, all designed to imitate team-sports movements. The same protocol was completed in two sessions, one with an assumed optimal geometrical setup of the LPS (optimal condition), and once with a sub-optimal geometrical setup of the LPS (sub-optimal condition). Raw two-dimensional position data were extracted from both the LPS and the reference system for accuracy assessment. Position, distance and speed were compared.

**Results:** The mean difference between the LPS and reference system for all position estimations was 0.21 ± 0.13 m (*n* = 30,166) in the optimal setup, and 1.79 ± 7.61 m (*n* = 22,799) in the sub-optimal setup. The average difference in distance was below 2% for all tasks in the optimal condition, while it was below 30% in the sub-optimal condition. Instantaneous speed showed the largest differences between the LPS and reference system of all variables, both in the optimal (≥35%) and sub-optimal condition (≥74%). The differences between the LPS and reference system in instantaneous speed were speed dependent, showing increased differences with increasing speed.

**Discussion:** Measures of position, distance, and average speed from the LPS show low errors, and can be used confidently in time-motion analyses for indoor team sports. The calculation of instantaneous speed from LPS raw data is not valid. To enhance instantaneous speed calculation the application of appropriate filtering techniques to enhance the validity of such data should be investigated. For all measures, the placement of anchor nodes and the field of play relative to the walls of the building influence LPS output to a large degree.

## Introduction

Analyses of physical demands can improve the understanding of physical performance and injury risk in sports. Such analyses are therefore conducted in many individual and team sports (Bangsbo et al., 2006; Montgomery et al., 2010; Gabbett, 2013; Gilgien et al., 2013; Luteberget and Spencer, 2017). In investigations of physical demands in team sports, the overall workload is often reported as a measure of athletes' total effort. Overall workload is dependent on the intensity and duration of the tasks, and is often reported using parameters such as total distance covered and distance covered in different speed zones. Sometimes high intensity events are also measured, which are characterized by inertia-based measures (Bangsbo et al., 2006; Michalsik et al., 2013; Luteberget and Spencer, 2017). High intensity events are reported using variables such as number of sprints, number of accelerations, or distances covered above a predefined speed threshold (Bangsbo et al., 2006; Michalsik et al., 2013; Luteberget and Spencer, 2017). To measure the parameters that describe these physical demands, Global Navigation Satellite Systems [GNSS; e.g., Global Positioning System (GPS)], inertial measurement units, a combination of the two, or video-based analysis systems are used. In outdoor sports, GNSS is one of the most frequently used methods for kinematic metrics in team sports (Malone et al., 2016). Total distance traveled, speed (e.g., time and distance in different speed zones), and number of sprints are calculated from position data, which can be obtained using GNSS technology, (sometimes integrated with inertial measurement units). The main drawback of GNSS is its restriction to outdoor facilities; therefore, indoor sports cannot use GNSS for tracking of players in competition and training. In indoor sports such as team handball, video-based analysis has been the main method used to analyze position-related variables (Sibila et al., 2004; Chelly et al., 2011; Michalsik et al., 2012, 2013; Póvoas et al., 2012, 2014; Karpan et al., 2015). However, in the past decade local positioning systems (LPSs) have been developed, which complement the role of hand operated and semi-automatic video based analysis systems in team sports (Leser et al., 2011). Most LPSs used in team sports are radio-frequency based (Muthukrishnan, 2009; Frencken et al., 2010; Ogris et al., 2012; Sathyan et al., 2012; Leser et al., 2014; Rhodes et al., 2014; Stevens et al., 2014), in which radio-frequency signals are used to measure the distance between several base stations (anchor nodes) at known locations distributed around the field of play, and mobile nodes worn by the athletes (Muthukrishnan, 2009; Hedley et al., 2010).

To allow meaningful analysis in sports, internal and external validity (Atkinson and Nevill, 2001) of systems used for data collection (e.g., LPS or GNSS) are important. External validity is related to the degree the data acquisition setting reflects the real sport setting. To maximize external validity, data acquisition should be conducted in a real-life sport setting, with minimal obstruction of the execution of the sport. Internal validity relates to the accuracy and repeatability of the measurements, and should be of a quality that allows quantification of small changes of practical importance within and between athlete activity profiles (Jennings et al., 2010). If the validity of a system is not sufficient, the implementation of training or competition results based on the measurement system may cause harm to athletes in terms of prescription of inadequate training, leading to decreased performance and/or increased health risks (Foster, 1998; Gabbett, 2004). In turn, this can result in reduced team performance, thus affecting a team's structure and economic situation. Compared with investigating athletes in a laboratory setting, external validity has been improved to a large degree by systems such as GNSS and LPS, as these facilitate data acquisition in real-life training and competition. However, optimization of external validity can have a negative impact on internal validity (Atkinson and Nevill, 2001). Thus, investigations of the accuracy and repeatability of systems are important in order to be confident about the validity of data.

The accuracy of GNSS has been quantified for use in individual sports (Waegli and Skaloud, 2009; Gilgien et al., 2013, 2014, 2015; Supej and Cuk, 2014; Boffi et al., 2016; Fasel et al., 2016; Specht and Szot, 2016) and for team sports over a wide range of courses and velocities (Coutts and Duffield, 2010; Jennings et al., 2010; Cummins et al., 2013; Johnston et al., 2013, 2014; Scott et al., 2016). However, to our knowledge, only a small number of studies have investigated the accuracy of LPS for team sports (Frencken et al., 2010; Ogris et al., 2012; Sathyan et al., 2012; Leser et al., 2014; Rhodes et al., 2014; Stevens et al., 2014). The accuracy of LPS is mainly dependent on the signal type; environmental conditions, such as obstructions and materials in the surroundings of the field of play; the geometry between signal anchor nodes and the units on the athletes (Muthukrishnan, 2009; Malone et al., 2016); and the signal analysis and parameter calculation process. Indoor venues have been shown to elicit greater errors in LPS compared to outdoor venues, probably as a consequence of an increased multipath propagation compared to outdoor conditions (Sathyan et al., 2012). Thus, validation of a positioning system should be executed in the typical conditions in which it is used. In GNSS, the geometrical setup of the satellites (anchor nodes) is outside the user's control. In LPS, on the other hand, the geometry of the anchor nodes can be altered by the user in the installation process. To our knowledge, no studies have assessed the effect of the anchor node setup and the positioning of the field of play relative to the building's walls (signal multipath problem) on the accuracy of LPS.

In commercial positioning systems, data processing, such as derivation of kinematic metrics from position data, may vary between different LPS and GNSS systems, and even between different software in the same service product (Gilgien et al., 2014; Malone et al., 2016). However, the derivation of metrics is often not elucidated in the manufacturer's documentation, which complicates comparisons between different systems and software (Malone et al., 2016; Specht and Szot, 2016). Currently multiple LPS systems are commercially available, which differ in data acquisition technology, sampling rates and data processing steps; this affects the validity of the data output (Malone et al., 2016; Varley et al., 2017). Thus, the validity of one system does not apply to other systems, and individual validation of each system is required.

The aim of the present study was to (1) determine the validity of position, distance traveled and instantaneous speed of a commercially available LPS (Catapult ClearSky T6, Catapult Sports, Australia) for indoor use; and (2) to investigate how the placement of the field of play relative to the anchor nodes and walls of the building affects the validity of the system. The study investigated these two questions in a typical indoor sport application, comparing the raw data from the LPS with a gold standard reference system (infrared light-based camera system).

## Method

In the present study, we investigated the validity of an LPS system for monitoring movements in indoor team-sport athletes. Two male and two female active team handball players [age, 23.0 ± 2.2 years; body mass, 76.6 ± 11.4 kg; height, 172.3 ± 10.1 cm; mean ± standard deviation (SD)] participated in the study. All participants received verbal and written information about the procedures of the study, and gave signed consent to participate in the study. The Norwegian Social Science Data Services approved the study.

### Data acquisition

**Figure 1:**
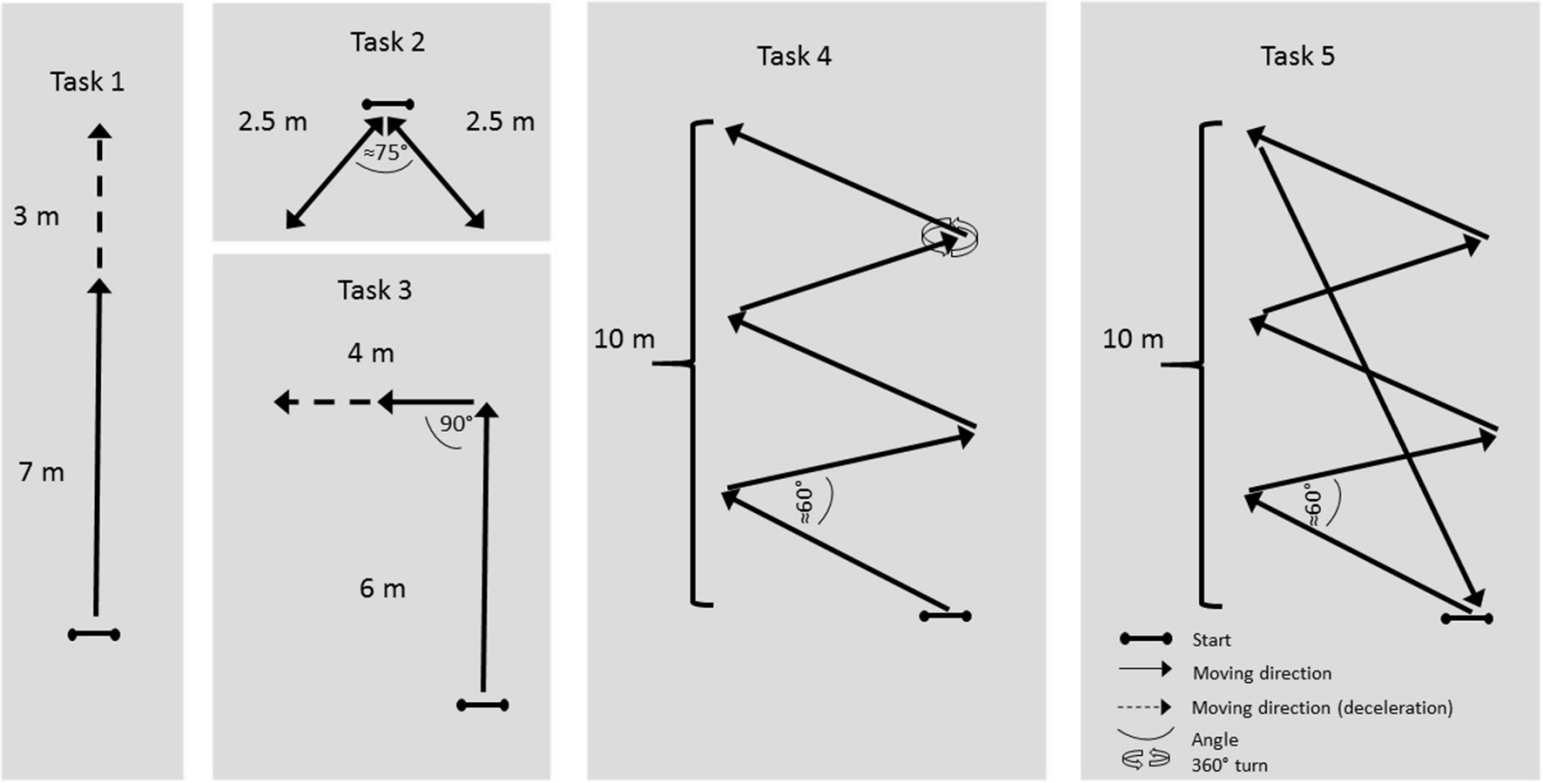
Diagram of the tasks.

**Figure 2:**
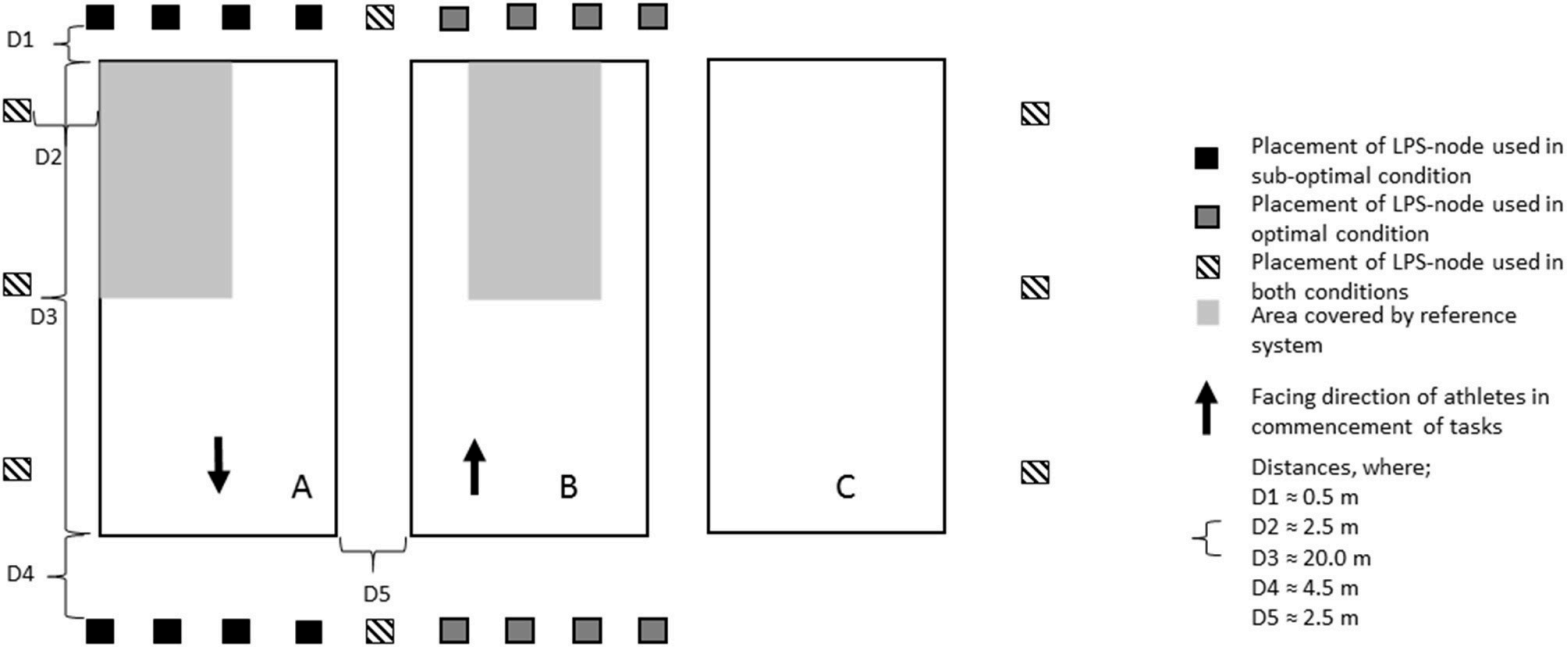
Setup of nodes around the handball court. The anchor nodes were suspended ~3 m above the floor.

The study was conducted in a sports hall measuring 50 × 70 × 11 m, on an indoor surface (Pulastic SP Combi, Gulv og Takteknikk AS, Norway). The participants completed a total of five tasks, all designed to imitate team-sports movements, as shown in Figure 1. Task 1: a straight-line sprint and deceleration to a stop. Task 2: two diagonal movements, forward and back to the left and the right, with the paths separated by an angle of ~75°.Task 3: a straight-line sprint, a 90° turn, and then deceleration to a stop. Task 4: a zig-zag (angle of turns ≈ 60°) course executed with sideways movements, and a 360° turn. Task 5: five continuous laps of the same course as in task 4, without the 360° turn. All tasks were commenced from a standing position. Each task was executed 5 times, with the exception of task 1, which was executed 9 times. Thus, a total of 116 trials were captured for each of the test conditions. Participants completed an individually selected warm-up before commencement of the tasks. All tasks were practiced during the warm-up. Participants were instructed to give maximal effort in all tasks. Subjects were tested on two separate days. The same protocol was completed in both sessions, on 1 day with an assumed optimal setup of the LPS (Optimal; Figure 1, field B), and on the other day with a sub-optimal setup of the LPS (Sub-optimal; Figure 1, field A). In the optimal setup, the LPS was arranged symmetrically, with a larger distance between the nodes and the testing area. In the sub-optimal setup, the LPS was asymmetrical, and the distance between the nodes and the testing area was small (Figure 2). This was done to replicate the effect of short distances between LPS anchor nodes and the field of play.

The LPS (Catapult ClearSky T6, Catapult Sports, Australia) and the reference system (Qualisys Oqus, Qualisys AB, Sweden) were installed around the field of play to capture the athletes' motion with both systems. During each trial 16 anchor nodes that were fixed around the handball court (Figure 2) collected LPS data, with a reported capturing frequency of 20 Hz. The LPS was set up to cover a field size of 20 × 40 m, the dimensions of an official team handball court. Each participant was instrumented with a lightweight (≈28 g) mobile node (firmware version: 1.40), measuring L: 40 mm × H: 52 mm × D: 14 mm. The mobile node was positioned between the shoulder blades, in the manufacturer-supplied vest (Catapult Sports, Australia). At all times during the data acquisition, 14 mobile nodes were turned on to simulate the usual data load on the system. The spatial calibration of the LPS was conducted using a tachymeter (Leica Builder 509 Total Station, Leica Geosystems AG, Switzerland), according to the manufacturer's recommendations preceding the testing sessions. Reference data was collected using eight infra-red cameras mounted on tripods around the testing area (Figure 2), using a capture frequency of 100 Hz. The capture volume was 10 × 14 m. A reflective marker, 12 mm in diameter, was mounted on the mobile node's center to obtain a three-dimensional position. The reference system was spatially calibrated according to the manufacturer's recommendations prior to the testing sessions. Infra-red camera systems, such as the reference system in this study, can provide accuracy within a possible error range in a magnitude of millimeters (Chiari et al., 2005; Windolf et al., 2008; Jensenius et al., 2012). The accuracy is dependent on the number of cameras used, capturing volume, technical specifications and settings of system parameters (Windolf et al., 2008; Jensenius et al., 2012). In the current study, the calibration was carried out using a calibration wand, with the exact length of 749.2 mm. The calibration resulted in a 6.14 mm and 6.85 mm SD of the wand length, for optimal and sub-optimal condition, respectively.

### Data processing

To compare the LPS-based data with the reference system, the coordinate system of the reference system was transformed into the LPS's coordinate system using a Helmert transformation (Sheynin, 1995). The transformation between the coordinate systems was based on four reference points (12 mm reflective markers, positioned 1 m above floor level, in the four corners of the testing area). The positions of the reference points were measured with the reference system in all trials, and with a tachymeter (Leica Builder 509 Total Station, Leica Geosystems AG, Switzerland) in the LPS coordinate system. The Helmert transformation resulted in a mean position residual per calibration point of 2.3 cm for the optimal condition and 0.4 cm for the sub-optimal condition.

Raw position data (X and Y coordinates) was extracted, both from the LPS and from the reference system, using their respective software (LPS: OpenField, Catapult Sports, Australia. Reference system: Qualisys Track Manager, Qualisys AB, Sweden). All data analyses were conducted in MatLab (The MathWorks inc., USA). Due to incomplete LPS raw data (resulting from loss of signal during parts of the trials), 22 (sub-optimal condition) and 1 (optimal condition) trials were excluded from further data analyses. The capture frequency of the LPS system was not constant. The mean capture frequency was calculated to be 17.5 Hz. To overcome the issue of a variable capture frequency, the position data, from both the LPS and reference system, were resampled at the mean capture frequency of the LPS using a second order natural spline function. Trials including data gaps >1 s were excluded from the analyses. This resulted in the exclusion of 30 (sub-optimal condition) and 12 (optimal condition) trials from analysis. Thus, 64 (55%) trials (sup-optimal condition) and 103 (89%) trials (optimal condition) were available for analysis in this study. LPS and reference system data were time synchronized using cross-correlation of speed data. For that purpose the following steps were undertaken: (1) Position data in the horizontal plane (X and Y coordinates) were differentiated to obtain horizontal plane speed, for both LPS and reference system, using a four-point finite central difference formula (Gilat and Subramaniam, 2011). (2) LPS and reference system data were time synchronized using cross-correlation (Buck et al., 2002) of horizontal plane speed data. After time synchronization, data was trimmed to reflect only the time athletes were performing the trials, by using a speed threshold of 0.5 m·s^−1^ (determined from the reference system). Two-dimensional position data at 17.5 Hz were used to calculate distance and speed. Distance traveled per trial was calculated as sum of the Euclidean distance between consecutive points. Speed in the horizontal plane (hereafter called speed) was calculated from position data, using a four-point finite central difference formula (Gilat and Subramaniam, 2011).

### Method comparison

The variables of position, distance and speed were compared for each task, using the norm of the differences between the LPS and the reference system. Mean difference, SD, and maximal difference in position were calculated. To express the results for position, the difference for each task from the reference system was assigned to bin limits in a histogram, and expressed as a percentage of the total number of raw data points, thus excluding the effect of duration of the task on the results. For distance, instantaneous and mean speed, the differences were characterized by mean, SD and maximal difference.

## Results

**Figure 3:**
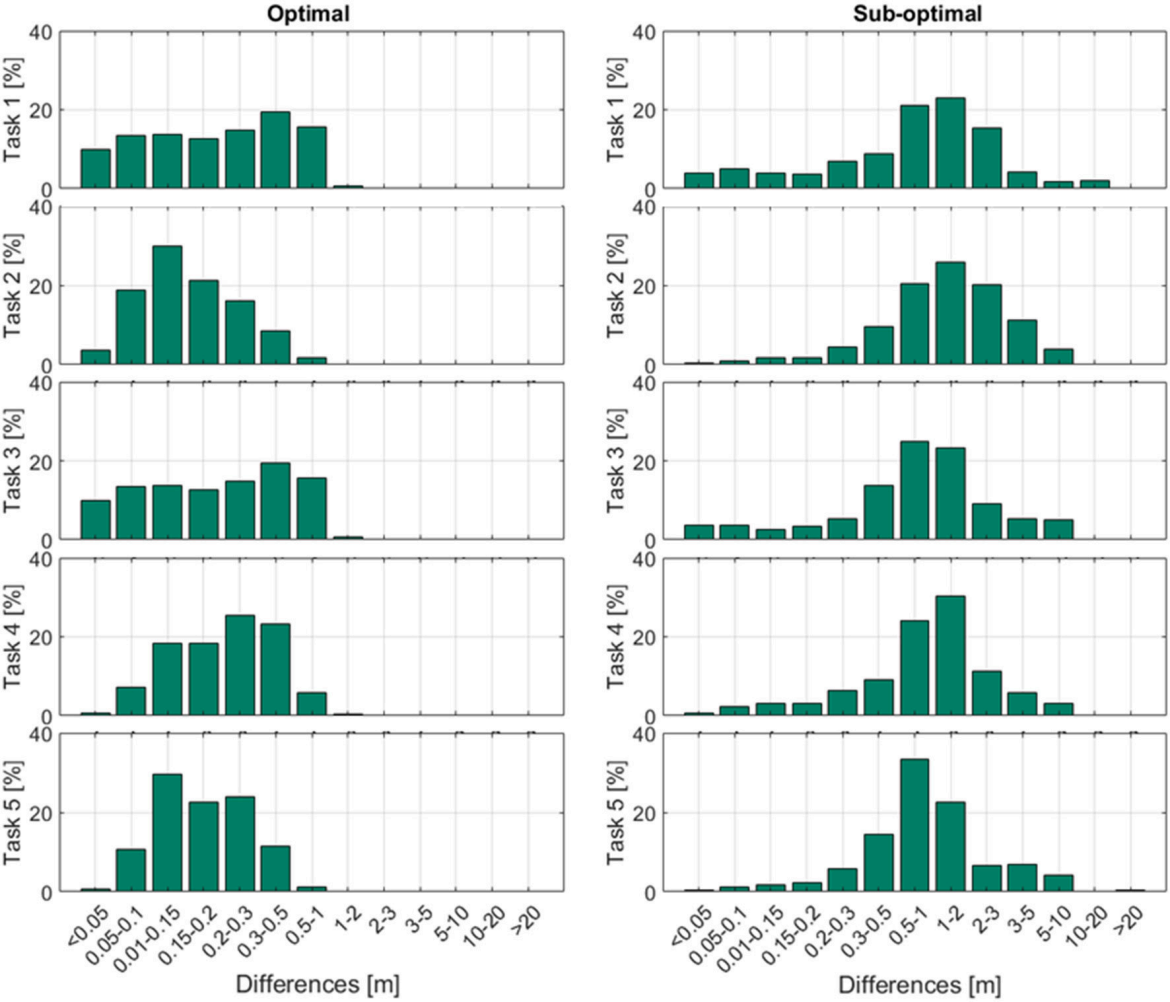
Distance differences for each task compared to the reference system. The differences were assigned to accuracy categories, and expressed as percentages of the total number of raw data points.

The mean difference between the LPS and reference system for all position estimations was 0.21 ± 0.13 m (*n* = 30′166) in the optimal setup, and 1.79 ± 7.61 m (*n* = 22′799) in the sub-optimal setup. Task 2 and task 5 showed the lowest mean (<0.20 m) and maximal differences (<1 m) in the optimal setup. In the sub-optimal condition, task 3 showed the lowest mean and maximal differences, but all differences in the sub-optimal condition were greater than in the optimal condition. Mean and maximum position differences for all tasks are displayed in Table 1. Figure 3 presents the difference distribution in position in the five tasks, for both the optimal and sub-optimal condition.

**Table 1:** Difference between the LPS and reference system for position, for optimal and sub-optimal condition respectively.

	**Optimal**			**Sub-optimal**		
	***n***	**Average (m)**	**Maximum (m)**	***n***	**Average (m)**	**Maximum (m)**
Task 1	2468	0.27 ± 0.22	1.40	1449	1.46 ± 1.95	13.07
Task 2	4675	0.17 ± 0.11	0.81	2822	1.72 ± 1.42	8.24
Task 3	1190	0.34 ± 0.24	1.41	565	1.37 ± 1.72	9.60
Task 4	2379	0.26 ± 0.17	1.91	2118	1.41 ± 1.52	9.85
Task 5	19454	0.19 ± 0.10	0.96	15845	1.89 ± 9.10	194.64

With respect to distance, the mean differences between systems were 0.31 ± 0.40 m and 11.42 ± 26.21 m in the optimal and sub-optimal condition, respectively, for all tasks combined. The mean difference was well below 2% in the optimal condition, for all tasks (Table 2). Task 5 showed the lowest difference in the optimal condition. In the sub-optimal condition, all tasks showed higher differences, of ≥15% in all tasks. The LPS overestimated the distance compared to the reference system for both the optimal and sub-optimal condition.

**Table 2:** Difference between the LPS and reference system for distance traveled, for optimal and sub-optimal condition respectively.

	**Optimal**						**Sub-optimal**					
	***n***	**Reference (m)**	**Average diff (m)**	**Max diff (m)**	**Average diff (%)**	**Max diff (%)**	***n***	**Reference (m)**	**Average diff (m)**	**Max diff (m)**	**Average diff (%)**	**Max diff (%)**
Task 1	34	9.52 ± 1.40	0.14 ± 0.26	1.00	1.5	10.5	17	9.90 ± 0.16	2.46 ± 2.10	7.68	24.9	77.6
Task 2	16	33.31 ± 1.25	0.60 ± 0.57	2.18	1.8	6.5	13	23.88 ± 1.53	6.92 ± 5.07	17.37	29.0	72.7
Task 3	19	9.41 ± 2.36	0.15 ± 0.21	0.86	1.6	9.1	8	11.71 ± 0.29	2.45 ± 2.75	8.73	20.9	74.5
Task 4	18	15.97 ± 6.19	0.24 ± 0.18	0.64	1.5	4.0	13	21.38 ± 2.47	3.21 ± 3.35	9.43	15.0	44.1
Task 5	16	132.81 ± 3.92	0.64 ± 0.46	1.65	0.5	1.2	13	140.17 ± 4.95	41.38 ± 48.23	192.54	29.5	137.4

**Figure 4:**
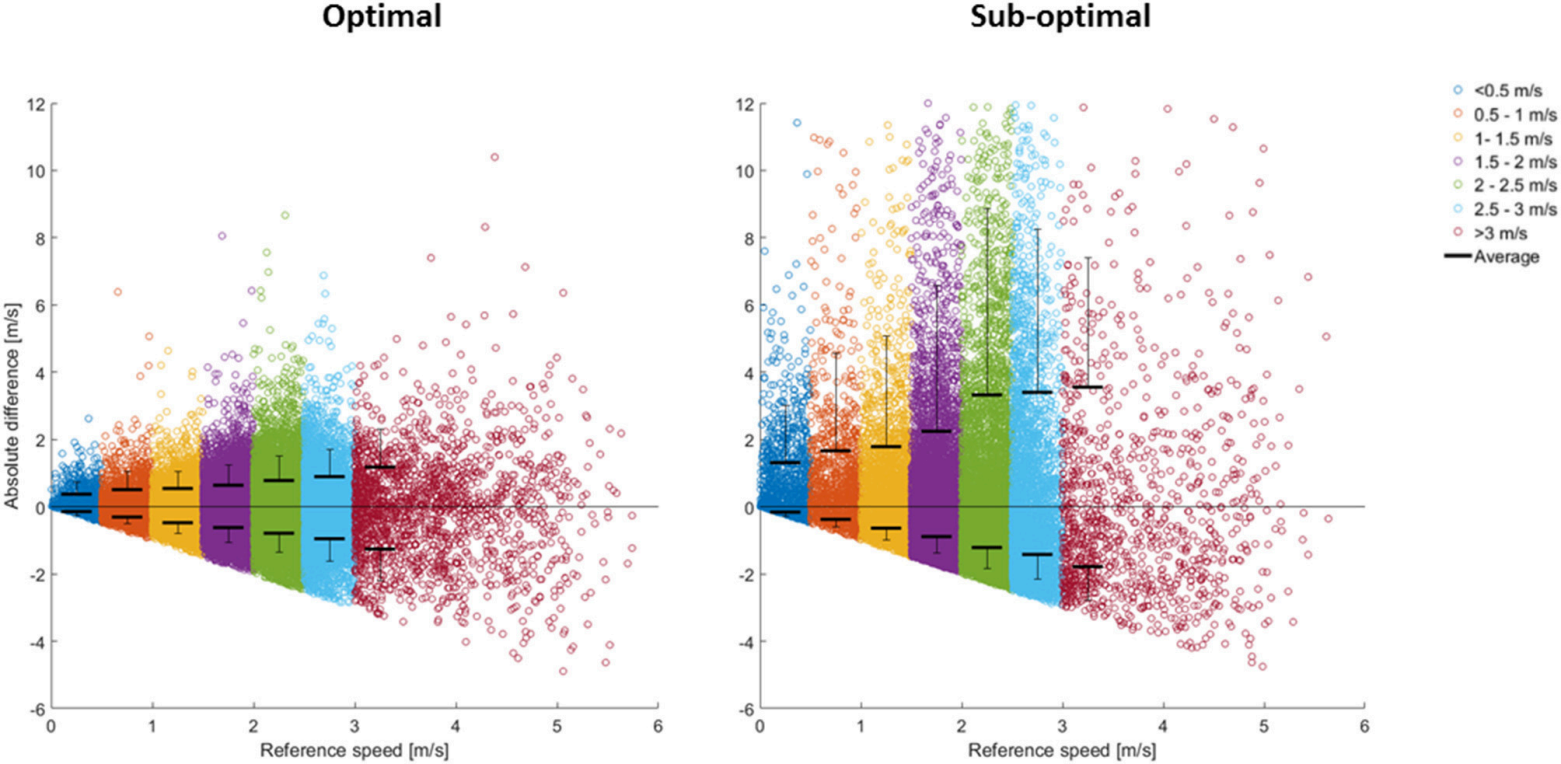
Differences in instantaneous speed from the reference system, divided into speed thresholds.

Instantaneous speed showed mean differences of ≥33% for both the optimal and sub-optimal condition (Table 3). Figure 4 displays all instantaneous speed measurements and reveals a direct association between speed and mean error. For mean speed, the mean difference was below 3% for all tasks (Table 4) in the optimal condition. The sub-optimal condition showed higher values across all tasks (≈15–30%).

**Table 3:** Difference between the LPS and reference system for instantaneous speed, for optimal and sub-optimal condition respectively.

	**Optimal**					**Sub-optimal**				
	***n***	**Average diff (m/s)**	**Max diff (m/s)**	**Average diff (%)**	**Max diff (%)**	***n***	**Average diff (m/s)**	**Max diff (m/s)**	**Average diff (%)**	**Max diff (%)**
Task 1	34	0.77 ± 0.86	10.40	34.8	375	17	1.43 ± 1.86	16.79	83.7	6101
Task 2	16	0.78 ± 0.70	7.56	33.5	237	13	1.60 ± 1.97	18.62	74.4	353
Task 3	19	0.92 ± 0.88	7.40	39.2	355	8	2.30 ± 2.94	31.25	87.7	982
Task 4	18	0.79 ± 0.71	8.10	35.3	477	13	1.64 ± 1.79	18.44	90.8	1175
Task 5	16	0.68 ± 0.58	8.67	37.0	197	13	1.73 ± 3.41	53.73	75.4	769

**Table 4:** Difference between the LPS and reference system for average speed, for optimal and sub-optimal condition respectively.

	**Optimal**						**Sub-optimal**					
	***n***	**Reference (m/s)**	**Average diff (m/s)**	**Max diff (m/s)**	**Average diff (%)**	**Max diff (%)**	***n***	**Reference (m/s)**	**Average diff (m/s)**	**Max diff (m/s)**	**Average diff (%)**	**Max diff (%)**
Task 1	34	2.30 ± 1.38	0.05 ± 0.14	0.77	2.2	33.3	17	1.93 ± 1.46	0.50 ± 0.47	2.02	26.0	105.1
Task 2	16	2.00 ± 0.71	0.03 ± 0.03	0.08	1.4	4.1	13	1.82 ± 0.76	0.50 ± 0.34	1.12	27.6	61.4
Task 3	19	2.64 ± 1.25	0.07 ± 0.17	0.71	2.8	26.9	8	2.75 ± 1.47	0.55 ± 0.62	2.00	20.2	72.8
Task 4	18	2.12 ± 0.79	0.05 ± 0.07	0.30	2.3	14.0	13	2.18 ± 0.90	0.32 ± 0.33	0.94	14.7	43.4
Task 5	16	1.91± 0.56	0.01 ± 0.01	0.02	0.5	1.2	13	1.90 ± 0.54	0.55 ± 0.67	2.65	29.1	139.0

## Discussion

The aim of the current study was to investigate the validity of a commercially available LPS designed to track indoor team sports. The mean difference in position between the LPS and the reference system was below 0.35 m in all tasks in the optimal condition, while in the sub-optimal condition the difference was above 8 m in all tasks. Mean difference in distance was below 2% in the optimal condition, while it was below 30% in the sub-optimal condition for all tasks. Instantaneous speed showed the largest differences between the LPS and reference systems of all measures tested, both in the optimal (≥35%) and sub-optimal condition (≥74%). Further, the difference between instantaneous speed measurement in the LPS and the reference system was dependent on the reference speed, with a higher speed yielding a higher difference.

The position error of LPS is often investigated with static measurements due to the lack of a reference system that allows instantaneous position comparisons in motion. Static measurements of the validity of LPS have shown an error range of ~1 to 32 cm (Frencken et al., 2010; Sathyan et al., 2012; Rhodes et al., 2014). This large range can partly be attributed to the different methodological setups and LPS technologies used. The largest error was found in an indoor environment (Rhodes et al., 2014), while the smallest error was found in an outdoor environment (Frencken et al., 2010). Only one previous study reported errors in position using LPS measurements in dynamic tasks, with a mean error of 0.23 m (Ogris et al., 2012). Although the previous reported value was from an outdoor environment, the results showed approximately the same error in position as in the optimal condition in the current study (0.21 m in the current study vs. 0.23 m in Ogris et al., 2012). Position measurements are mainly used for time motion analyses in sports, and thus our results seem acceptable for this purpose. However, for other applications, such as tactical analyses, the lack of information regarding the accuracy level needed makes it difficult to confidently state that the LPS is either acceptable or not. The similarity in error between the outdoor study by (Ogris et al., 2012) and the current indoor study could indicate that measurements in large halls with no obstructions may create measurement conditions that are not much different from outdoor conditions. However, the current study also seems to indicate that small distances to walls and corners of halls, along with the anchor node setup, have a major impact on position accuracy.

Previous studies on LPS in indoor conditions show mean errors ranging from 2.0 to 3.5% (Sathyan et al., 2012; Leser et al., 2014), while studies in outdoor conditions have shown errors ranging from 0.2 to 3.9% (Frencken et al., 2010; Sathyan et al., 2012; Stevens et al., 2014). Presumably, previous studies optimized the setup of the LPS when investigating the accuracy of the systems, resembling the optimal condition in the current study. The results of the current study showed a mean difference in distance from the reference system of between 0.5 and 1.8% in the optimal condition, which is lower than previously reported for indoor conditions. Some previous studies showed an underestimation of distance with LPS systems (Frencken et al., 2010; Leser et al., 2014; Stevens et al., 2014), while others overestimated distance (Sathyan et al., 2012; Rhodes et al., 2014). The studies that showed an overestimation of distance were conducted indoors, as was the current study, leading to the speculation that indoor conditions may be a contributing factor to the overestimation. However, the differences could also be caused by differences in the filtering techniques applied in different studies (Sathyan et al., 2012). In the current study, no filters were applied to the data, in order to investigate the raw output from the LPS. Further investigations of the effect of filtering techniques on the validity of the current data could be interesting, as filtering techniques can affect the estimated distance and speed (Sathyan et al., 2012; Malone et al., 2016). Distance traveled might be less vulnerable to position error, since no amplification of error through position derivation of position was conducted, as was done with speed. However, error in distance traveled in sub-optimal conditions was of a critically large magnitude, and not useful for quantifying the distance covered for training load purposes. Hence, for quantification of distance, only data from the optimal condition can be used with confidence. In addition, it might be reasonable to investigate whether filtering techniques could reduce the error in distance for sub-optimal conditions.

To our knowledge, very few studies have investigated the validity of instantaneous speed measurements in team sports (Varley et al., 2012). However, in match and training analyses, distance data are often categorized into speed zones in order to provide a more comprehensive metric for “intensity distribution” of the athletes external loading (Malone et al., 2016). Such categorization relies on instantaneous speed measurements. It has been previously shown that peak speeds in LPS are less accurate than mean speeds (Ogris et al., 2012; Rhodes et al., 2014; Stevens et al., 2014); however, no previous study has assessed the accuracy of instantaneous speed as determined with an LPS over the whole range of dynamic tasks in team sports. The current study shows that instantaneous speed differed substantially between LPS and the reference system in both the optimal and sub-optimal condition (Table 4), and that the differences were speed-dependent (Figure 4). Our study shows considerably higher errors than those previously shown in a GNSS study (Varley et al., 2012). However, the GNSS-based study investigated straight line running only, which could contribute to these results. In addition, time synchronization and filtering of raw data could play a significant role in error reduction for instantaneous speed (Ogris et al., 2012; Stevens et al., 2014), and the filtering techniques and time synchronization method used in the aforementioned study (Varley et al., 2012) were not disclosed. Mean speed has been investigated in several studies (Frencken et al., 2010; Ogris et al., 2012; Rhodes et al., 2014; Stevens et al., 2014), and is often used as an overall indicator of the intensity of an activity. Compared to previous studies, the current study shows similar results (Table 3) in terms of mean speed errors (Frencken et al., 2010; Ogris et al., 2012; Rhodes et al., 2014; Stevens et al., 2014), thus, the LPS can give an overall indication of the intensity of the activity.

In the current study, the same measurement system was applied with the same measurement setting, but in two different conditions (optimal and sub-optimal condition). The factors that changed between the two conditions were the anchor node positions relative to the field of play and the distance between the side walls and corners of the hall to the field of play. The current study shows that changes in the placement of anchor node positions relative to the field of play and the distance between the side walls and corners of the hall to the field of play can affect the accuracy of data. Placement of nodes has an effect on the geometry of the anchor nodes relative to each other and the mobile node. In addition to changes in geometry, close proximity of the edge of the field and the walls may cause the mobile nodes to go undetected by multiple anchor nodes, thus producing a higher error rate. Close proximity between the edge of the field and the walls may also increase multipath propagation (Muthukrishnan, 2009), which will reduce the accuracy of data. The current study was not designed to isolate the different contributors (geometry, undetected nodes, and multipath propagation), thus the results of this study show the sum of errors accumulated from all sources. Further investigations are needed to understand the impact of the different contributors and how this could contribute to the optimization of anchor node placement.

## Limitations

The method used in this study resulted in a position difference of 2.3 and 0.4 cm between the LPS and reference system, during optimal and sub optimal conditionings respectively. This is sufficient to detect the differences between the systems.

The effect of anchor node placement is especially important in smaller sports halls, when all distances to the walls are small. In the current study, both conditions were tested in a large sports hall, in order to keep variables such as distance to ceiling and material of walls and floors constant. The current results for the sub-optimal setup cannot be assumed to be true for smaller sports halls, since small sport halls will have shorter distances between field of play and the walls on all four sides of the field, while in the current study only two side walls were close to the field of play. In small sports halls we might therefore expect even higher errors than in the sub-optimal condition of the current study. However, the study showed that changing the anchor node positions relative to the field of play and the distance between the side walls and corners of the hall to the field of play does affect the accuracy of the system. To optimize the measurement setup in small sport halls, future investigations should include tilting of nodes in the vertical direction to the field of play, and optimization of the geometry of anchor node positions relative to the field of play. Special attention should be given to multipath minimization to avoid mobile nodes going undetected by multiple anchor nodes close to corners by adjusting the tilting and positioning of nodes close to corners.

In the current study the raw positional data was examined. However, not all systems provide unfiltered raw positioning data for the user. In addition, practitioners will most likely not process data in independent software. Hence, validation of software-derived metrics is still needed, and should also be undertaken in future for the system investigated in this study. The current study provides insight into the raw positional data and the errors in the acquisition technology, without the possible influence of the manufacturer's software, which is important for researchers who want to process data using independent software. The export of raw positioning data from the systems allows filtering and processing of metrics independent of the manufacturer's software. Using manufacturer-independent software for raw data treatment and metric calculation may not only increase control of the process (Malone et al., 2016), but also avoid inaccuracies when collecting longitudinal data, which will be affected by software updates and other changes in the capture system. In addition, independent processing allows the user to provide details on the data processing in publications to facilitate appropriate interpretations and ease replication by other investigators. The positioning data (granted that it is not subjected to any filtering) is not affected by software updates, and thus could be used as a more stable measure of validity than software-derived metrics. In addition, raw position might be the most unaffected variable and should be used as the primary variable to compare measurements between different positioning systems' acquisition technology.

## Conclusions and practical applications

The accuracy of LPS output is highly sensitive to relative positioning between field of play and walls/corners and anchor nodes. Measures of position, distance, and mean speed from the LPS can be used confidently in time-motion analyses for indoor team sports, in conditions similar to the optimal condition in this study. In small sport halls or in conditions when walls, and especially the corners of the room are close to the field of play, accuracy is relatively poor and caution is indicated.

The LPS is not valid in calculating instantaneous speed from raw data. Therefore the use of LPS systems for quantifying distance covered at different velocity bands is not recommended. The application of appropriate filtering techniques to enhance the validity of such data should be investigated.

Future studies should assess the relative contribution to total error of (1) signal multipath effects, which occur to a larger extent in close proximity to walls and corners; and (2) by the positioning and orientation of anchor nodes relative to the field of play. The inclusion of a dilution of precision measure would enhance the optimization of anchor node positions.

## Ethics statement

This study was carried out in accordance with the recommendations of Regional Comitees for Medicine and Health Research Etichs with written informed consent from all subjects. All subjects gave written informed consent in accordance with the Declaration of Helsinki. The studys data storage methods was approved by the Norwegian Social Science Data Service.

## Author contributions

LL, MS and MG conceptualized the study design. LL and MG conducted the data acquisition. LL and MG contributed to the analysis of data, and all authors contributed to the interpretation of the data. LL drafted the manuscript, and all other authors revised it critically. All authors approved the final version and agreed to be accountable for all aspects of this work.

### Conflict of interest statement

The authors declare that the research was conducted in the absence of any commercial or financial relationships that could be construed as a potential conflict of interest.
